# Low Skeletal Muscle Index as a Predictor of Pathological Complete Response in HER-2 Positive and Triple-Negative Breast Cancer

**DOI:** 10.3390/medicina61091508

**Published:** 2025-08-22

**Authors:** Murat Günaltılı, Murad Guliyev, Mehmet Cem Fidan, Zeliha Birsin, Emir Çerme, Vali Aliyev, Hamza Abbasov, Selin Cebeci, Seda Jeral, Özkan Alan, Nebi Serkan Demirci, Çiğdem Papila, Onur Erdem Şahin, Said Erkam Bıyıkoğlu, Tülin Öztürk, Berrin Papila

**Affiliations:** 1 Division of Medical Oncology, Department of Internal Medicine, Cerrahpaşa Faculty of Medicine, Istanbul University-Cerrahpaşa, Istanbul 34098, Turkey; drmuradguliyev@gmail.com (M.G.); mcemfidan@hotmail.com (M.C.F.); zelihabirsin@gmail.com (Z.B.); emircrm34@gmail.com (E.Ç.); dktr.aliyev@gmail.com (V.A.); hamzaabbasov90@gmail.com (H.A.); sellcebeci@gmail.com (S.C.); sedajeral@gmail.com (S.J.); ozkan.alan@hotmail.com (Ö.A.); drserkannebi@yahoo.com (N.S.D.); bpapila@iuc.edu.tr (Ç.P.); 2 Department of Nuclear Medicine, Cerrahpaşa Faculty of Medicine, Istanbul University-Cerrahpaşa, Istanbul 34098, Turkey; onur.sahin@iuc.edu.tr (O.E.Ş.); said.biyikoglu@iuc.edu.tr (S.E.B.); 3 Department of Pathology, Cerrahpaşa Faculty of Medicine, Istanbul University-Cerrahpaşa, Istanbul 34098, Turkey; drozturk@iuc.edu.tr; 4 Department of General Surgery, Cerrahpaşa Faculty of Medicine, Istanbul University-Cerrahpaşa, Istanbul 34098, Turkey

**Keywords:** breast cancer, sarcopenia, pathological response, SMI

## Abstract

*Background and Objectives:* Breast cancer is a leading cause of cancer-related mortality, particularly in aggressive subtypes such as HER2-positive and triple-negative breast cancer (TNBC). Achieving a pathological complete response (pCR) after neoadjuvant therapy is strongly associated with improved survival outcomes in these subgroups, making the prediction of pCR a clinical priority. Sarcopenia, a progressive loss of skeletal muscle mass and strength, is increasingly recognized in cancer patients and has been linked to chemotherapy toxicity and poorer survival. However, its specific impact on pCR in HER2-positive and TNBC patients remains unclear. This study aimed to evaluate the association between radiologically defined sarcopenia, or a low skeletal muscle index (SMI), and pathological response in these subtypes, and to explore its potential as a predictive biomarker. *Materials and Methods:* This retrospective study included patients with HER2-positive or TNBC who received neoadjuvant therapy between January 2015 and October 2023. SMI was assessed using pre-treatment positron emission tomography images at the L3 vertebral level, with values < 38.5 cm^2^/m^2^ considered as low. Univariate and multivariate logistic regression analyses were performed to identify factors associated with pCR. *Results:* A total of 85 patients were included, with low SMI present in 35 (41.2%). In univariate analysis, clinical stage and low SMI were associated with pCR. However, in the multivariate model, only low SMI remained an independent predictor. Patients without low SMI had higher odds of achieving pCR (odds ratio [OR] 4.13; 95% confidence interval [CI] 1.55–10.95; *p* = 0.004). Low SMI was also associated with higher rates of treatment-related toxicity (42.9% vs. 20.0%, *p* = 0.023). *Conclusions:* Pre-treatment low SMI is strongly associated with lower pCR rates in patients with HER2-positive and TNBC undergoing neoadjuvant therapy. These findings underscore the importance of early identification and management of radiologically defined sarcopenia to optimize treatment response and improve clinical outcomes.

## 1. Introduction

Breast cancer is the most commonly diagnosed malignancy among women and remains one of the leading causes of cancer-related mortality worldwide [1,2]. The prognosis and response to treatment vary significantly across molecular subtypes. In particular, HER2-positive and triple-negative breast cancer (TNBC) subtypes exhibit more aggressive behavior and are associated with worse outcomes [3]. However, with advancements in systemic therapy, survival for these subtypes has improved considerably. One of the most important contributors to this improvement is the achievement of pathological complete response (pCR) after neoadjuvant therapy. In both HER2-positive and TNBC patients, pCR is strongly associated with longer disease-free survival (DFS) and overall survival (OS) [4,5]. Consequently, identifying predictive markers of pCR is essential for optimizing individualized treatment strategies [6,7].

Sarcopenia, a condition defined by progressive loss of skeletal muscle mass and function due to aging or chronic disease, is increasingly recognized in patients with cancer [8]. It can be diagnosed using dual-energy X-ray absorptiometry (DEXA), bioelectrical impedance analysis (BIA), or computed tomography (CT) [9]. DEXA, although commonly used, lacks the ability to assess intramuscular fat, limiting its diagnostic accuracy [10]. In contrast, CT allows for the evaluation of lean muscle area with greater precision, especially when skeletal muscle index (SMI) is measured at the third lumbar vertebra (L3), a site known for its sensitivity and reproducibility [11,12]. Although limited, emerging data suggest that SMI measurements using positron emission tomography (PET)-CT and PET-magnetic resonance imaging (MRI), modalities routinely employed in cancer staging and follow-up, can also be used to assess sarcopenia [13,14,15].

In oncological research, sarcopenia is most frequently assessed using imaging-based methods. Cross-sectional imaging at the L3 level provides a reproducible and well-validated approach to quantify SMI, and this radiological method has been widely adopted in cancer studies. Accordingly, radiologically defined sarcopenia, based on low SMI derived from imaging, offers a practical and objective measure that allows for consistent evaluation across studies and has become an established surrogate marker in oncological research [12,13,16].

Because achieving pCR is a critical goal in HER2-positive and TNBC subtypes, identifying reliable predictors of treatment response is clinically valuable. Several factors—such as tumor grade, hormone receptor status, Ki-67 index, tumor size, choice of neoadjuvant regimen, and BRCA mutation status—have been associated with pCR rates [17,18,19,20,21]. Radiologically defined sarcopenia, which has been linked to increased chemotherapy toxicity and poorer survival across various malignancies, may also influence the likelihood of achieving pCR [22]. Several biological mechanisms have been suggested in the literature to explain this association. Chronic systemic inflammation, frequently observed in cancer patients, can accelerate muscle protein breakdown and exacerbate skeletal muscle loss, which in turn may impair chemotherapy tolerance. A reduction in muscle mass is also linked to diminished availability of essential amino acids necessary for immune cell proliferation and function, potentially weakening anti-tumor immune responses. Moreover, alterations in body composition can influence the pharmacokinetics of chemotherapeutic agents by modifying their volume of distribution and clearance, which may increase toxicity risk and compromise optimal dose intensity [23,24,25]. Nevertheless, this relationship remains insufficiently studied in HER2-positive and TNBC patients. Interestingly, some studies in operable breast cancer populations have reported a possible positive correlation between radiologically defined sarcopenia and pCR [26].

Given that achieving pCR in HER2-positive and TNBC patients is strongly associated with improved outcomes, clarifying the impact of low SMI on the treatment response in this context is critical. This study aimed to evaluate the association between radiologically defined sarcopenia, assessed as low SMI on PET-based imaging, and pCR in patients with HER2-positive and TNBC undergoing neoadjuvant therapy. We also sought to explore the potential of low SMI as a novel biomarker for predicting treatment response in these patient groups.

## 2. Materials and Methods

### 2.1. Patients and Data Collection

This retrospective study included 85 patients diagnosed with HER2-positive or triple-negative breast cancer who received neoadjuvant therapy between January 2015 and October 2023. All participants had histopathologically confirmed breast cancer who also had radiologically verified early or locally advanced stage disease. Diagnosis was established through core needle biopsy or fine-needle aspiration, and all specimens were reviewed by an experienced breast pathologist at our center prior to the initiation of neoadjuvant chemotherapy. After completion of neoadjuvant therapy, all patients underwent surgical resection. Treatment planning was conducted by a multidisciplinary tumor board in accordance with national and international clinical guidelines, and the decision to administer neoadjuvant chemotherapy, including the selection of specific regimens, was based on each patient’s clinicopathological profile. Demographic, clinical, and histopathological data were retrospectively collected from the hospital records. The evaluated parameters included age, clinical stage at diagnosis, tumor grade, histological subtype, hormone receptor status (in HER2-positive patients), Ki-67 index, body mass index (BMI, weight in kilograms divided by the square of height in meters), pre-treatment skeletal muscle area (SMA), skeletal muscle index (SMI), baseline laboratory parameters (including neutrophil count, lymphocyte count, hemoglobin, C-reactive protein, and albumin), PET response, pathological complete response (pCR) status, and Miller-Payne grade. Pathological complete response was defined as the absence of invasive cancer in both the breast and axillary lymph nodes (ypT0/Tis, ypN0). Tumor response was evaluated using the Miller-Payne grading system [27].

Patients without pre-treatment PET imaging, those who experienced disease progression during neoadjuvant therapy, those unable to undergo surgery, and those who did not complete their entire neoadjuvant systemic treatment at our institution were excluded, as the primary objective was to assess the relationship between low SMI and pCR.

### 2.2. Assessment of Body Composition and Definition of Low SMI

**Figure 1:**
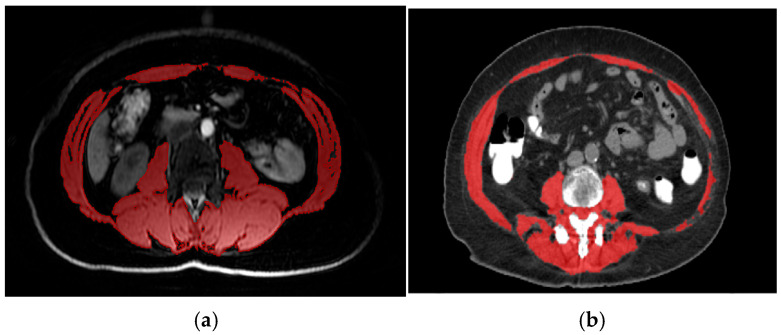
(**a**) Axial PET-MRI slice at the L3 vertebra in a patient with triple-negative breast cancer. Skeletal muscle area (red) was manually segmented on T1-weighted, water-only images using 3D Slicer (version 5.7.0; www.slicer.org, accessed on 13 May 2024; The Slicer Community, Brigham and Women’s Hospital, Boston, MA, USA) based on anatomical landmarks and signal intensity. SMI was calculated by dividing the muscle cross-sectional area (cm^2^) by height squared (m^2^). (**b**) Axial PET-CT slice at the L3 vertebra in a patient with triple-negative breast cancer. Skeletal muscle area (red) was segmented using CoreSlicer (version 1.0.0; Montreal, QC, Canada) with predefined Hounsfield unit thresholds (–29 to +150 HU). SMI was calculated as total skeletal muscle area (cm^2^) divided by height squared (m^2^). White areas in both images represent contrast enhancement.

SMI was assessed using PET-CT or PET-MRI images obtained prior to neoadjuvant therapy. It was calculated at the level of the third lumbar vertebra (L3) by dividing the total skeletal muscle area (cm^2^) by the square of the patient’s height (m^2^) (Figure 1). To establish an optimal threshold for low SMI, a receiver operating characteristic (ROC) analysis was performed; however, no clearly discriminative cut-off with sufficient sensitivity and specificity was identified. Therefore, a cut-off value of <38.5 cm^2^/m^2^ was used to define radiologically defined sarcopenia, in accordance with previously validated criteria for women [12,28].

All SMI measurements were performed using PET-CT or PET-MRI images acquired with the same imaging device for all patients. Manual segmentation at the L3 level was independently conducted by two nuclear medicine specialists, both blinded to patient outcomes. All cases were subsequently reviewed together to reach consensus and minimize variability in segmentation.

In the overall cohort, besides radiologically defined sarcopenia, the presence of sarcopenic obesity was also evaluated. Sarcopenic obesity was defined as the coexistence of radiologically defined sarcopenia (based on the SMI cut-off described above) and obesity (BMI ≥ 30 kg/m^2^) [29]. In addition to the separate assessment of radiologically defined sarcopenia and BMI, sarcopenic obesity was analyzed as an independent parameter.

### 2.3. Statistical Analysis

Statistical analyses were performed using SPSS version 26 (IBM Corp., Armonk, NY, USA). Continuous variables were expressed as median (range) and compared using the Mann–Whitney U test or Student’s *t*-test, as appropriate for the distribution of the data. Categorical variables were reported as frequencies and percentages, and compared using the chi-square test or Fisher’s exact test. Logistic regression analyses were conducted to identify factors associated with pCR, with odds ratios (ORs) and 95% confidence intervals (CIs) reported. To determine an optimal cut-off value for the presence of low SMI, a receiver operating characteristic (ROC) analysis was also performed.

In accordance with recommendations in the literature, variables with *p* < 0.25 in univariate analysis, together with variables considered clinically relevant based on prior evidence, were entered into the multivariate logistic regression model using backward stepwise selection. This approach enables the inclusion of factors that may not reach statistical significance in univariate testing but could become significant when evaluated in combination with other variables, particularly those representing potential confounders or having established clinical relevance [30]. All statistical tests were two-sided, and a *p*-value < 0.05 was considered statistically significant.

## 3. Results

### 3.1. Characteristics of Patients

This study included 85 patients with breast cancer who received neoadjuvant treatment. The median age was 49 years (range: 24–77). Of the total cohort, 49 patients (56.6%) had HER2-positive disease, and 36 (42.4%) had triple-negative breast cancer. The vast majority (84.7%) had invasive ductal carcinoma.

SMI was assessed using PET-CT in 44 (52%) patients and PET-MRI in 41 (48%) patients. Low SMI was identified in 35 patients (41.2%) before the initiation of neoadjuvant therapy. Among the baseline characteristics, BMI was the only variable that differed significantly between the groups. The low SMI group had a significantly lower median BMI compared to the normal SMI (25.8 vs. 29.5 kg/m^2^, *p* = 0.003).

No statistically significant differences were observed between low SMI and normal SMI patients in terms of menopausal status, histologic type, molecular subtype, tumor grade, Ki-67 index, clinical T and N stage, clinical TNM stage, or neoadjuvant treatment regimens (all *p* > 0.05). The baseline characteristics according to SMI status are summarized in Table 1.

### 3.2. Pathological Complete Response Outcomes

pCR was achieved in 45 patients (52.9%) after neoadjuvant therapy. In the HER2-positive group, pCR was observed in 31 of 49 patients (63.3%) and in 14 of 36 patients in the TNBC group (38.9%). The difference between the two groups was statistically significant (*p* = 0.026). Post-treatment pCR was achieved in 34 of 50 (68%) normal SMI patients compared with 11 of 35 (31.4%) low SMI patients, demonstrating a statistically significant difference (*p* < 0.001).

To evaluate the factors associated with achieving a pCR, both univariate and multivariate logistic regression analyses were performed.

In the univariate analysis, several factors were found to be significantly associated with pCR. Patients with clinical T1–2 tumors had higher odds of achieving pCR compared to those with T3–4 tumors (odds ratio [OR] 2.66, 95% confidence interval [CI] 1.01–7.01, *p* = 0.047). Similarly, patients with clinical N0 status were more likely to achieve pCR than those with N1–3 disease (OR 3.66, 95% CI 1.08–12.35, *p* = 0.037). Notably, the presence of low SMI was a strong predictor of pCR. Patients with normal SMI had significantly higher odds of achieving pCR compared to patients with low SMI (OR 4.64, 95% CI 1.83–11.73, *p* = 0.001). Clinical stage was not statistically significant, although stage II patients had higher pCR rates than those with stage III disease (OR 2.37, 95% CI 0.98–5.74, *p* = 0.055). In addition, hormone receptor (HR) status, histologic grade, and Ki-67 index met the predefined inclusion criterion for the multivariate model (*p* < 0.25), as specified in the Methods section, even though they did not reach statistical significance in univariate testing (*p* = 0.118, *p* = 0.130, and *p* = 0.209, respectively). Other factors, including age, menopausal status, and BMI, were not significantly associated with pCR in univariate analysis.

In the multivariate analysis adjusting for potential confounding variables, radiologically defined sarcopenia (low SMI) remained the only independent predictor of pCR. Patients with normal SMI had a significantly increased likelihood of achieving pCR (OR 5.17, 95% CI 1.74–15.40, *p* = 0.003). Variables that were significant in the univariate model, such as the clinical T stage, N stage, and overall stage, did not retain statistical significance in the multivariate analysis (Table 2).

Sarcopenic obesity, defined as the coexistence of obesity (BMI ≥ 30 kg/m^2^) and radiologically defined sarcopenia, was present in 8 patients (9.4%) in the study cohort. pCR was achieved in 2 of 8 patients (25.0%) with sarcopenic obesity and in 43 of 77 patients (55.8%) without sarcopenic obesity. The difference between the two groups did not reach statistical significance (Fisher’s exact test, *p* = 0.140).

### 3.3. Treatment-Related Toxicity Outcomes

The association between low SMI and treatment-related toxicity was also analyzed. Grade 2 or higher toxicity was observed in 15 of 35 patients with low SMI (42.9%) compared to 10 of 50 patients with normal SMI (20%) (*p* = 0.023). Among the patients who developed toxicity, hematologic adverse events, including anemia, neutropenia, and thrombocytopenia, were reported in 17 of 25 patients (68%).

Grade ≥ 2 non-hematologic adverse events occurred in 8 patients (32%), consisting of neuropathy (n = 4), fatigue (n = 3), and urinary tract infection (n = 1). Treatment delays due to toxicity were observed in 18 of 85 patients (21.2%) in the entire cohort. These occurred in 10 patients (28.6%) in the low SMI group and 8 patients (14.5%) in the normal SMI group, with no statistically significant difference between groups (*p* = 0.163).

## 4. Discussion

Radiologically defined sarcopenia is a common and clinically significant condition in cancer patients, resulting from multifactorial causes such as chronic inflammation, malnutrition, treatment-related toxicities, and decreased physical activity. It has been linked to adverse outcomes, including increased toxicity, reduced treatment efficacy, and inferior survival across various malignancies [12,31,32]. While sarcopenia has been extensively studied in breast cancer, its association with pathological response remains unclear. In this study, we demonstrated that pre-treatment radiologically defined sarcopenia (low SMI) was strongly associated with reduced pathological complete response rates in patients with HER2-positive and triple-negative breast cancer receiving neoadjuvant therapy.

The prevalence of radiologically defined sarcopenia in our cohort was 41.2%, which was slightly higher than that reported in previous breast cancer studies. A meta-analysis by Roberto et al. found radiologically defined sarcopenia in 33% of patients (n = 6130) and a separate analysis by Dai et al. reported a prevalence of 38% [33,34]. In another pooled analysis by Simmons et al., the rate was 36.3% among non-metastatic patients, increasing to 55.1% in the metastatic setting [35]. Differences in patient selection, tumor stage, treatment exposure, and definitions of sarcopenia may explain the variability in reported prevalence. Notably, Karaca et al. showed an increase in radiologically defined sarcopenia prevalence from 24.8% to 40.7% after neoadjuvant treatment, highlighting the dynamic nature of sarcopenia during therapy [36].

In our study, BMI was significantly lower in patients with low SMI. This finding aligns with Caan et al., who reported lower BMI in radiologically defined sarcopenic patients (24.7 vs. 30.0 kg/m^2^) [23]. However, Karaca et al. reported that radiologically defined sarcopenic patients had BMIs over 30 [36]. This discrepancy may reflect differences in sarcopenia measurement methods or patient characteristics. Importantly, BMI alone is not a reliable surrogate for muscle mass. Sarcopenia can occur at any BMI level and body composition analysis remains the gold standard for accurate diagnosis [26,33,37].

Several studies have confirmed the negative impact of radiologically defined sarcopenia on survival outcomes in breast cancer [34,38,39]. However, its relationship with pCR remains inconclusive. While our findings demonstrated a strong negative association between low SMI and pCR (OR 4.13, 95% CI 1.55–10.95), other studies have reported conflicting results.

Contrary to our findings, in a retrospective study by Del Fabbro et al. [26] involving 129 patients with breast cancer, radiologically defined sarcopenia was found to potentially increase the likelihood of achieving pCR; however, this relationship was not statistically significant (OR 2.74, 95% CI 0.92–8.22, *p* = 0.071). However, in this study, only 18 of 129 patients (13.9%) had radiologically defined sarcopenia, and it is highly likely that the low rate of sarcopenic patients may have affected the results. In contrast, Karaca et al. reported that non-sarcopenic patients had significantly higher pCR rates (*p* = 0.012), although radiologically defined sarcopenia itself was not an independent predictor [36]. Similarly, Isiklar et al. observed lower pCR rates in radiologically defined sarcopenic patients, but the association was not statistically significant [40]. In a TNBC-specific analysis, Guo et al. found that pre-treatment radiologically defined sarcopenia was a significant independent predictor of treatment response [41].

These inconsistencies are likely due to differences in study design, sample size, patient subtypes, sarcopenia measurement techniques, and cut-off thresholds. Despite these limitations, our findings highlight the potential of radiologically defined sarcopenia as a clinically relevant predictor of pCR, especially in HER2-positive and TNBC subtypes, where achieving pCR has clear prognostic value.

In our analysis of sarcopenic obesity, patients with this condition exhibited lower pCR rates compared to those without, although the difference did not reach statistical significance. This likely reflects the limited statistical power of this small subgroup. Few studies have directly evaluated the clinical implications of sarcopenic obesity in breast cancer and most available evidence comes from heterogeneous cancer populations. Meta-analyses and observational studies in these settings have reported associations between sarcopenic obesity and poorer overall, recurrence-free, and disease-free survival, as well as higher rates of postoperative complications, prolonged hospitalization, and reduced functional status [12,29,42]. Collectively, these findings suggest that sarcopenic obesity appears to represent an adverse prognostic factor in oncology, warranting further investigation in breast cancer patients receiving neoadjuvant therapy.

Similar to many other cancers, the association between radiologically defined sarcopenia and treatment-related toxicity in breast cancer has been demonstrated in numerous studies and meta-analyses [13,22,32,43]. In a meta-analysis by Roberto et al. [33], the risk of developing grade 3–4 toxicity was higher in the radiologically defined sarcopenic group (42%) than in the non-sarcopenic group (19%) (OR 3.58, 95% CI 2.11–6.06, *p* < 0.0001). Jang et al. [44], in their meta-analysis evaluating 9863 breast cancer patients, found that radiologically defined sarcopenia was associated with both increased rates of treatment-related toxicity and dose reductions or discontinuation of treatment. Our study corroborates these findings, showing significantly more frequent grade ≥ 2 toxicities in patients with low SMI (42.9% vs. 20%, *p* = 0.023). These data underscore the need for early radiologically defined sarcopenia identification and supportive interventions such as nutritional therapy and physical activity to enhance treatment tolerance and completion rates. İn this context, recent evidence on trace element homeostasis has shown an association with breast cancer development in BRCA1 mutation carriers. Zinc and copper are key regulators of redox homeostasis, influencing oxidative stress levels, which in turn can affect skeletal muscle metabolism and integrity [45]. Considering that nutritional status can influence both trace element balance and sarcopenia, it is plausible that similar mechanisms may also impact treatment response and toxicity in breast cancer.

Although CT-based skeletal muscle measurement at the L3 level is widely accepted for radiologically defined sarcopenia diagnosis [12,32,43,44], we utilized PET-CT and PET-MRI in our study. While literature supporting the use of PET for this purpose remains limited in breast cancer, prior studies have shown a high correlation between PET and CT for assessing body composition [13,14,15]. In an observational study conducted by Caan et al. with 3241 breast cancer patients, PET-CT was used for body composition assessment to diagnose sarcopenia [23]. Albano et al. reported consistent results between standard CT and PET-derived measurements, supporting the validity of PET-based assessment [14].

This study has several limitations. First, the retrospective design and relatively small sample size may introduce selection bias, limit the generalizability of the findings, and constrain the statistical power of subgroup analyses. Second, our study was based solely on imaging-derived SMI measurements. Although radiologically defined sarcopenia is commonly applied in oncological research, this approach does not capture functional parameters such as muscle strength or physical performance, which are essential to the clinical definition of sarcopenia. In addition, the use of PET-based SMI measurements, while promising, has not yet been standardized or validated for radiologically defined sarcopenia diagnosis in breast cancer populations. Third, we did not assess changes in radiologically defined sarcopenia status over the course of treatment, as post-treatment PET imaging was not routinely performed for all patients in our center, which precluded the evaluation of longitudinal changes in SMI and their potential impact on treatment efficacy. Finally, other potentially relevant factors influencing SMI, such as nutritional status and physical activity levels, could not be evaluated due to a lack of available data.

Prospective validation of the prognostic role of radiologically defined sarcopenia in larger, multicenter cohorts is needed, as current evidence is limited by heterogeneous measurement methods and varying cut-off values across studies. In addition, interventional approaches to prevent or reverse low SMI warrant further investigation. Structured resistance and aerobic exercise programs, combined with targeted nutritional strategies such as individualized dietary counseling and supplementation, have demonstrated potential benefits in maintaining or restoring muscle mass, improving treatment tolerance, and enhancing oncologic outcomes [33,46].

## 5. Conclusions

In conclusion, our study demonstrates that low SMI is an independent negative predictor of pathological complete response in patients with HER2-positive and TNBC receiving neoadjuvant therapy. These findings highlight the importance of early identification and management of radiologically defined sarcopenia in this population. Further prospective studies are warranted to validate these results and explore targeted interventions that may enhance treatment outcomes in patients with low SMI.

## Figures and Tables

**Table 1:** Baseline Characteristics of Patients Stratified by SMI Status.

Variables	All Patients n = 85 (%)	Normal SMI n = 50 (%)	Low SMI n = 35 (%)	*p*
**Age (years)**				0.409
Median (min–max)	49 (24–77)	49 (29–71)	47 (24–77)	
**BMI (kg/m^2^)**				0.003
Median (min–max)	28.3 (18.3–44.3)	29.5 (20.0–44.3)	25.8 (18.3–38.9)	
**Menopausal Status, n (%)**				0.713
Premenopausal	49 (57.6)	28 (56.0)	21 (60.0)	
Postmenopausal	36 (42.4)	22 (44.0)	14 (40.0)	
**Histological type, n (%)**				0.918
Invasive ductal carcinoma	72 (84.7)	43 (86.0)	29 (82.9)	
Invasive lobular carcinoma	2 (2.4)	1 (2.0)	1 (2.9)	
Others	11 (12.9)	6 (12.0)	5 (14.3)	
**Molecular subtype, n (%)**				0.682
HR+, HER-2 +	33 (38.8)	21 (42.0)	12 (34.3)	
HR−, HER-2 +	16 (18.8)	8 (16.0)	8 (22.9)	
Triple negative	36 (42.4)	21 (42.0)	15 (42.9)	
**Histologic grade, n (%)**				0.814
Grade 1–2	26 (30.6)	16 (32.0)	10 (28.6)	
Grade 3	59 (69.4)	34 (68.0)	25 (71.4)	
**Ki-67 (%)**				0.635
Median (min–max)	45 (5–90)	45 (5–90)	40 (15–90)	
**cT Stage, n (%)**				0.622
T1	6 (7.1)	4 (8.0)	2 (5.7)	
T2	54 (63.5)	33 (66.0)	21 (60.0)	
T3	13 (15.3)	8 (16.0)	5 (14.3)	
T4	12 (14.1)	5 (10.0)	7 (20.0)	
**cN Stage, n (%)**				0.160
N0	17 (20.0)	14 (28.0)	3 (8.6)	
N1	33 (38.8)	18 (36.0)	15 (42.9)	
N2	20 (23.5)	11 (22.0)	9 (25.7)	
N3	15 (17.6)	7 (14.0)	8 (22.9)	
**Clinical TNM Stage, n (%)**				0.320
Stage II	37 (43.5)	24 (48.0)	13 (37.1)	
Stage III	48 (56.5)	26 (52.0)	22 (62.9)	
**Radiological evaluation method, n (%)**				0.169
PET-CT	44 (51.8)	29 (58.0)	15 (42.9)	
PET-MRI	41 (48.2)	21 (42.0)	20 (57.1)	
**Surgery, n (%)**				0.804
BCS	28 (32.9)	17 (34.0)	11 (31.4)	
Mastectomy	57 (67.1)	33 (66.0)	24 (68.6)	
Neoadjuvant treatment regimen, n (%) *				0.236
Anthracycline and taxane based with trastuzumab	15 (30.6)	7 (24.1)	8 (40.0)	
Anthracycline and taxane based with trastuzumab and pertuzumab	34 (69.4)	22 (75.9)	12 (60.0)	
Neoadjuvant treatment regimen, n (%) **				0.955
Anthracycline and taxane based without platinum	19 (52.8)	11 (52.4)	8 (53.3)	
Anthracycline and taxane based with platinum	17 (47.2)	10 (47.6)	7 (46.7)	

* HER2-positive patients only. ** TNBC patients only. BMI: body mass index; HR: hormone receptor; HER2: human epidermal growth factor receptor 2; TNBC: triple-negative breast cancer; BCS: breast-conserving surgery; PET-CT: positron emission tomography/computed tomography; PET-MRI: positron emission tomography/magnetic resonance imaging; cT stage: clinical tumor stage; cN stage: clinical nodal stage.

**Table 2:** Univariate and Multivariate Logistic Regression Analyses of Factors Associated with Pathological Complete Response (pCR).

Variables		Pathologic Response		Univariate Analysis		Multivariate Analysis	
		CR n = 45 (%)	Non-CR n = 40 (%)	OR (95% CI)	*p*	OR (95% CI)	*p*
**Age (years)**	<49	23 (56.1)	18 (43.9)	Ref.			
	≥49	22 (50.0)	22 (50.0)	0.78 (0.33–1.84)	0.574		
**Menopausal Status**	Pre	27 (55.1)	22 (44.9)	Ref.			
	Post	18 (50.0)	18 (50.0)	0.81 (0.34–1.93)	0.642		
**BMI (kg/m^2^)**	<30	30 (53.6)	26 (46.4)	Ref.			
	≥30	15 (51.7)	14 (48.3)	0.93 (0.38–2.28)	0.871		
**HR status**	Negative	24 (46.2)	28 (53.8)	Ref			
	Positive	21 (63.6)	12 (36.4)	2.04 (0.83–4.99)	0.118	1.29 (0.43–3.93)	0.648
**Grade**	Grade 1–2	17 (65.4)	9 (34.6)	Ref.			
	Grade 3	28 (47.5)	31 (52.5)	0.48 (0.18–1.24)	0.130	0.57 (0.18–1.79)	0.340
**Ki 67**	<45	23 (60.5)	15 (39.5)	Ref.			
	≥45	22 (46.8)	25 (53.2)	0.57 (0.24–1.37)	0.209	0.42 (0.14–1.30)	0.132
**cT Stage**	T3–4	9 (36.0)	16 (64.0)	Ref.			
	T1–2	36 (60.0)	24 (40.0)	2.66 (1.01–7.01)	0.047	3.08 (0.95–10.07)	0.062
**cN Stage**	N1–3	32 (47.1)	36 (52.9)	Ref.			
	N0	13 (76.5)	4 (23.5)	3.66 (1.08–12.35)	0.037	1.84 (0.38–8.95)	0.450
**Clinical Stage**	Stage III	21 (43.8)	27 (56.2)	Ref.			
	Stage II	24 (64.9)	13 (35.1)	2.37 (0.98–5.74)	0.055	1.06 (0.28–3.97)	0.936
**Low SMI**	Yes	11 (31.4)	24 (68.6)	Ref.			
	No	34 (68.0)	16 (32.0)	4.64 (1.83–11.73)	0.001	5.17 (1.74–15.40)	0.003

HR: hormone receptor; OR: odds ratio; CI: confidence interval; pCR: pathological complete response; BMI: body mass index; cT stage: clinical tumor stage; cN stage: clinical nodal stage; Ref.: reference.

## Data Availability

The datasets generated and/or analyzed during the current study are available from the corresponding author.

## Abbreviations

The following abbreviations are used in this manuscript:

BCS	Breast-conserving surgery
BIA	Bioelectrical impedance analysis
BMI	Body mass index
CI	Confidence interval
cN stage	Clinical nodal stage
cTstage	Clinical tumor stage
CT	Computed tomography
DFS	Disease-free survival
DEXA	Dual-energy X-ray absorptiometry
HER-2	Human epidermal growth factor receptor-2
HR	Hormone receptor
HU	Hounsfield unit
L3	Third lumbar vertebra
MRI	Magnetic resonance imaging
OR	Odds ratio
OS	Overall survival
pCR	Pathological complete response
PET	Positron Emission Tomography
PET-CT	Positron Emission Tomography/Computed Tomography
PET-MRI	Positron Emission Tomography/Magnetic resonance imaging
ROC	Receiver Operating Characteristic
SMA	Skeletal Muscle Area
SMI	Skeletal Muscle Index
TNBC	Triple-Negative Breast Cancer

## References

[1] Sung H., Ferlay J., Siegel R.L., Laversanne M., Soerjomataram I., Jemal A., Bray F. (2021). Global cancer statistics 2020: GLOBOCAN estimates of incidence and mortality worldwide for 36 cancers in 185 countries. CA Cancer J. Clin..

[2] Siegel R.L., Miller K.D., Fuchs H.E., Jemal A. (2022). Cancer statistics, 2022. CA Cancer J. Clin..

[3] Harbeck N., Gnant M. (2017). Breast cancer. Lancet.

[4] Denkert C., Von Minckwitz G., Darb-Esfahani S., Lederer B., Heppner B.I., Weber K.E., Budczies J., Huober J., Klauschen F., Furlanetto J. (2018). Tumour-infiltrating lymphocytes and prognosis in different subtypes of breast cancer: A pooled analysis of 3771 patients treated with neoadjuvant therapy. Lancet Oncol..

[5] Haque W., Verma V., Hatch S., Klimberg V.S., Butler E.B., Teh B.S. (2018). Response rates and pathologic complete response by breast cancer molecular subtype following neoadjuvant chemotherapy. Breast Cancer Res. Treat..

[6] Spring L.M., Fell G., Arfe A., Sharma C., Greenup R., Reynolds K.L., Smith B.L., Alexander B., Moy B., Isakoff S.J. (2020). Pathologic complete response after neoadjuvant chemotherapy and impact on breast cancer recurrence and survival: A comprehensive meta-analysis. Clin. Cancer Res..

[7] Guliyev M., Alan Ö., Günaltılı M., Safarov S., Fidan M.C., Şen G.A., Değerli E., Papila B., Demirci N.S., Papila Ç. (2024). Obesity is an independent prognostic factor that reduced pathological complete response in operable breast cancer patients. Medicina.

[8] Cruz-Jentoft A.J., Bahat G., Bauer J., Boirie Y., Bruyère O., Cederholm T., Cooper C., Landi F., Rolland Y., Sayer A.A. (2019). Sarcopenia: Revised European consensus on definition and diagnosis. Age Ageing.

[9] Cesari M., Landi F., Vellas B., Bernabei R., Marzetti E. (2014). Sarcopenia and physical frailty: Two sides of the same coin. Front. Aging Neurosci..

[10] Plank L.D. (2005). Dual-energy X-ray absorptiometry and body composition. Curr. Opin. Clin. Nutr. Metab. Care.

[11] Hansen R.D., Williamson D.A., Finnegan T.P., Lloyd B.D., Grady J.N., Diamond T.H., Smith E.U., Stavrinos T.M., Thompson M.W., Gwinn T.H. (2007). Estimation of thigh muscle cross-sectional area by dual-energy X-ray absorptiometry in frail elderly patients. Am. J. Clin. Nutr..

[12] Prado C.M., Lieffers J.R., McCargar L.J., Reiman T., Sawyer M.B., Martin L., Baracos V.E. (2008). Prevalence and clinical implications of sarcopenic obesity in patients with solid tumours of the respiratory and gastrointestinal tracts: A population-based study. Lancet Oncol..

[13] Albano D., Pasinetti N., Dondi F., Giubbini R., Tucci A., Bertagna F. (2022). Prognostic role of pre-treatment metabolic parameters and sarcopenia derived by 2-[18F]-FDG PET/CT in elderly mantle cell lymphoma. J. Clin. Med..

[14] Albano D., Camoni L., Rinaldi R., Tucci A., Zilioli V.R., Muzi C., Ravanelli M., Farina D., Coppola A., Camalori M. (2021). Comparison between skeletal muscle and adipose tissue measurements with high-dose CT and low-dose attenuation correction CT of 18F-FDG PET/CT in elderly Hodgkin lymphoma patients: A two-centre validation. Br. J. Radiol..

[15] Yuan H., Tan X., Sun X., He L., Li D., Jiang L. (2023). Role of 18F-FDG PET/CT and sarcopenia in untreated non-small cell lung cancer with advanced stage. Jpn. J. Radiol..

[16] Zwart A.T., Kok L.M.C., de Vries J., van Kester M.S., Dierckx R.A.J.O., de Bock G.H., van der Hoorn A., Halmos G.B. (2023). Radiologically Defined Sarcopenia as a Biomarker for Frailty and Malnutrition in Head and Neck Skin Cancer Patients. J. Clin. Med..

[17] Orsaria P., Grasso A., Ippolito E., Pantano F., Sammarra M., Altomare C., Cagli B., Costa F., Perrone G., Soponaru G. (2021). Clinical outcomes among major breast cancer subtypes after neoadjuvant chemotherapy: Impact on breast cancer recurrence and survival. Anticancer Res..

[18] Zhao B., Zhao H., Zhao J. (2018). Impact of hormone receptor status on the efficacy of HER2-targeted treatment. Endocr.-Relat. Cancer.

[19] Boughey J.C., McCall L.M., Ballman K.V., Mittendorf E.A., Ahrendt G.M., Wilke L.G., Taback B., Leitch A.M., Flippo-Morton T., Hunt K.K. (2014). Tumor biology correlates with rates of breast-conserving surgery and pathologic complete response after neoadjuvant chemotherapy for breast cancer: Findings from the ACOSOG Z1071 (Alliance) Prospective Multicenter Clinical Trial. Ann. Surg..

[20] Tan Q.-X., Qin Q.-H., Yang W.-P., Mo Q.-G., Wei C.-Y. (2014). Prognostic value of Ki67 expression in HR-negative breast cancer before and after neoadjuvant chemotherapy. Int. J. Clin. Exp. Pathol..

[21] Wunderle M., Gass P., Häberle L., Flesch V.M., Rauh C., Bani M.R., Hack C.C., Schrauder M.G., Jud S.M., Emons J. (2018). BRCA mutations and their influence on pathological complete response and prognosis in a clinical cohort of neoadjuvantly treated breast cancer patients. Breast Cancer Res. Treat..

[22] Shachar S.S., Deal A.M., Weinberg M., Nyrop K.A., Williams G.R., Nishijima T.F., Benbow J.M., Muss H.B. (2017). Skeletal muscle measures as predictors of toxicity, hospitalization, and survival in patients with metastatic breast cancer receiving taxane-based chemotherapy. Clin. Cancer Res..

[23] Caan B.J., Feliciano E.M.C., Prado C.M., Alexeeff S., Kroenke C.H., Bradshaw P., Quesenberry C.P., Weltzien E.K., Castillo A.L., Olobatuyi T.A. (2018). Association of muscle and adiposity measured by computed tomography with survival in patients with nonmetastatic breast cancer. JAMA Oncol..

[24] Bellieni A., Fusco D., Sanchez A.M., Franceschini G., Di Capua B., Allocca E., Di Stasio E., Marazzi F., Tagliaferri L., Masetti R. (2021). Different impact of definitions of sarcopenia in defining frailty status in a population of older women with early breast cancer. J. Pers. Med..

[25] Bilen M.A., Martini D.J., Liu Y., Shabto J.M., Brown J.T., Williams M., Khan A.I., Speak A., Lewis C., Collins H. (2020). Combined Effect of Sarcopenia and Systemic Inflammation on Survival in Patients with Advanced Stage Cancer Treated with Immunotherapy. Oncologist.

[26] Del Fabbro E., Parsons H., Warneke C.L., Pulivarthi K., Litton J.K., Dev R., Palla S.L., Brewster A., Bruera E. (2012). The relationship between body composition and response to neoadjuvant chemotherapy in women with operable breast cancer. Oncologist.

[27] Ogston K.N., Miller I.D., Payne S., Hutcheon A.W., Sarkar T.K., Smith I., Schofield A., Heys S.D. (2003). A new histological grading system to assess response of breast cancers to primary chemotherapy: Prognostic significance and survival. Breast.

[28] Gu D.H., Kim M.Y., Seo Y.S., Kim S.G., Lee H.A., Kim T.H., Jung Y.K., Kandemir A., Kim J.H., An H. (2018). Clinical usefulness of psoas muscle thickness for the diagnosis of sarcopenia in patients with liver cirrhosis. Clin. Mol. Hepatol..

[29] Yip C., Dinkel C., Mahajan A., Siddique M., Cook G., Goh V. (2015). Imaging body composition in cancer patients: Visceral obesity, sarcopenia and sarcopenic obesity may impact on clinical outcome. Insights Imaging.

[30] Bursac Z., Gauss C.H., Williams D.K., Hosmer D.W. (2008). Purposeful selection of variables in logistic regression. Source Code Biol. Med..

[31] Findlay M., White K., Lai M., Luo D., Bauer J.D. (2020). The Association Between Computed Tomography-Defined Sarcopenia and Outcomes in Adult Patients Undergoing Radiotherapy of Curative Intent for Head and Neck Cancer: A Systematic Review. J. Acad. Nutr. Diet..

[32] Prado C.M., Baracos V.E., McCargar L.J., Reiman T., Mourtzakis M., Tonkin K., Mackey J.R., Koski S., Pituskin E., Sawyer M.B. (2009). Sarcopenia as a determinant of chemotherapy toxicity and time to tumor progression in metastatic breast cancer patients receiving capecitabine treatment. Clin. Cancer Res..

[33] Roberto M., Barchiesi G., Resuli B., Verrico M., Speranza I., Cristofani L., Pediconi F., Tomao F., Botticelli A., Santini D. (2024). Sarcopenia in Breast Cancer Patients: A Systematic Review and Meta-Analysis. Cancers.

[34] Dai Y., Lan J., Li S., Xu G. (2024). Exploring the impact of sarcopenia on mortality in breast cancer patients: A comprehensive systematic review and meta-analysis. Breast Care.

[35] Simmons L.O., Cagney D., Hassan F., Lim J.Y., O’leary D.P., Liew A., Redmond H.P., Corrigan M., O’sullivan M., Kelly L. (2019). Prevalence of sarcopenia and its impact on survival in breast cancer—A systematic review and meta-analysis. Mesentery Peritoneum.

[36] Karaca M., Alemdar M.S., Karaca Ö.D., Kılar Y., Köker G., Sözel H., Yıldız M., Köker G.Ö., Arici M.Ö. (2024). Sarcopenia’s Role in Neoadjuvant Chemotherapy Outcomes for Locally Advanced Breast Cancer: A Retrospective Analysis. Med. Sci. Monit..

[37] Sun X., Xu J., Chen X., Zhang W., Chen W., Zhu C., Sun J., Yang X., Wang X., Hu Y. (2020). Sarcopenia in patients with normal body mass index is an independent predictor for postoperative complication and long-term survival in gastric cancer. Clin. Transl. Sci..

[38] Du L., Liu X., Zhu Q., Zhu K., Li P. (2024). Sarcopenia as a prognostic factor and multimodal interventions in breast cancer. Int. J. Gen. Med..

[39] Zhang X., Dou Q., Zeng Y., Yang Y., Cheng A., Zhang W. (2020). Sarcopenia as a predictor of mortality in women with breast cancer: A meta-analysis and systematic review. BMC Cancer.

[40] Isıklar A., Yilmaz E., Basaran G. (2024). The Relationship Between Body Composition and Pathological Response to Neoadjuvant Chemotherapy in Breast Cancer Patients. Cureus.

[41] Guo J., Meng W., Li Q., Zheng Y., Yin H., Liu Y., Zhao S., Ma J. (2024). Pretreatment sarcopenia and MRI-based radiomics to predict the response of neoadjuvant chemotherapy in triple-negative breast cancer. Bioengineering.

[42] Gao Q., Hu K., Gao J., Shang Y., Mei F., Zhao L., Chen F., Ma B. (2022). Prevalence and prognostic value of sarcopenic obesity in patients with cancer: A systematic review and meta-analysis. Nutrition.

[43] Aleixo G.F.P., Williams G.R., Nyrop K.A., Muss H.B., Shachar S.S. (2019). Muscle composition and outcomes in patients with breast cancer: Meta-analysis and systematic review. Breast Cancer Res. Treat..

[44] Jang M.K., Park S., Raszewski R., Park C.G., Doorenbos A.Z., Kim S. (2024). Prevalence and clinical implications of sarcopenia in breast cancer: A systematic review and meta-analysis. Support. Care Cancer.

[45] Matuszczak M., Kiljańczyk A., Marciniak W., Derkacz R., Stempa K., Baszuk P., Bryśkiewicz M., Cybulski C., Dębniak T., Gronwald J. (2024). Antioxidant Properties of Zinc and Copper-Blood Zinc-to Copper-Ratio as a Marker of Cancer Risk BRCA1 Mutation Carriers. Antioxidants.

[46] Deluche E., Lachatre D., Di Palma M., Simon H., Tissot V., Vansteene D., Meingan P., Mohebi A., Lenczner G., Pigneur F. (2022). Is sarcopenia a missed factor in the management of patients with metastatic breast cancer?. Breast.

